# Mental and physical health outcomes of war migrants

**DOI:** 10.1192/j.eurpsy.2023.26

**Published:** 2023-07-19

**Authors:** U. A. Valdimarsdóttir

**Affiliations:** Centre of Public Health Sciences, University of Iceland, Reykjavik, Iceland

## Abstract

**Abstract:**

According to the UN Refugee Agency, around 100 million individuals were estimated to be forcibly displaced worldwide in 2022 due to violence, war or other human rights violations. This represents a doubling in the global population of forcibly displaced individuals during the last decade. War migrants are frequently exposed to multiple traumas, including direct or indirect exposure to violence, as well as extended periods of insecurities in access to housing, clothes, food, education and health care. As a corollary, war migrants face extensive health risks, including elevated risks of psychiatric disorders with potential implications for the development of other conditions, e.g. autoimmune and cardiovascular diseases. The most recent evidence of the health risks of war migrants will be reviewed along with possible mitigating factors of such adverse sequalae.

**Disclosure of Interest:**

None Declared

